# Direct and indirect dorsolateral striatum pathways reinforce different action strategies

**DOI:** 10.1016/j.cub.2016.02.036

**Published:** 2016-04-04

**Authors:** Ana M. Vicente, Pedro Galvão-Ferreira, Fatuel Tecuapetla, Rui M. Costa

**Affiliations:** 1 Champalimaud Neuroscience Programme, Champalimaud Centre for the Unknown, Lisbon, Portugal

## Abstract

The basal ganglia, and the striatum in particular, are critical for action reinforcement 1, 2. The dorsal striatum, which can be further subdivided into dorsomedial (DMS) and dorsolateral (DLS) striatum, is mainly composed of two subpopulations of striatal medium spiny projection neurons (MSNs): dopamine D1 receptor-expressing MSNs that constitute the striatonigral or direct pathway (dMSNs); and dopamine D2 receptor-expressing MSNs that constitute the striatopallidal or indirect pathway (iMSNs) [3]. It has been suggested that each pathway has opposing roles in reinforcement, with dMSNs being important to learn positive reinforcement and iMSNs to learn to avoid undesired actions (Go/No-Go) [1]. Furthermore, optogenetic self-stimulation of dMSNs in DMS leads to reinforcement of actions, while self-stimulation of iMSNs leads to avoidance of actions [2]. However, in DLS, which has been implicated in the consolidation of well-trained actions and habits in mice 4, 5, both pathways are active during lever-pressing for reward [6]. Furthermore, extensive skill training leads to long-lasting potentiation of glutamatergic inputs into both dMSNs and iMSNs [4]. We report here that, in DLS, both dMSNs and iMSNs are involved in positive reinforcement, but support different action strategies.

## Teaser

It has been postulated that striatal direct and indirect projection pathways have opposing roles in action reinforcement. Vicente *et al.* show that, in dorsolateral striatum, optogenetic self-stimulation of each pathway can support positive reinforcement, but promotes the development of different action strategies.

## Main Text

To investigate the role of DLS striatonigral and striatopallidal neurons in action reinforcement, we used a self-stimulation paradigm where we activated specifically each pathway upon lever-pressing. We used a viral Cre-dependent approach to express Channelrhodopsin-2 (ChR) in either dMSNs (D1-Cre) or iMSNs (D2-Cre) of DLS (Supplemental Figure S1A). Mice were then trained in an operant box with two levers (Supplemental Figure S1B): an active lever where pressing resulted in the delivery of blue light (473 nm), and an inactive lever (no light delivered). Reinforced lever presses resulted in the delivery of light into DLS (2 seconds, 5 Hz, 10 ms wide pulses, frequency similar to the endogenous activity of MSNs [7]) (Supplemental Figure S2A,B). Each session lasted 30 minutes with no maximum number of reinforcers. Both groups of ChR-expressing mice increased the number of presses with training, and pressed significantly more than YFP controls (Supplemental Figure S2C, D1-cre, F1,10 = 20.67, P = 0.0011; D2-cre, F1,17 = 5.845, P = 0.0271).

**Figure 1:**
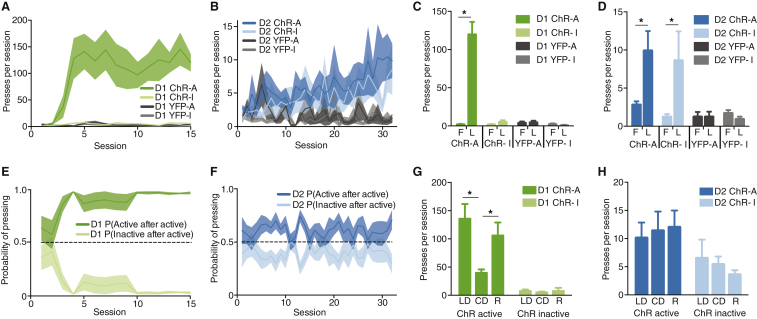
Optogenetic self-stimulation of striatonigral and striatopallidal DLS neurons supports the reinforcement of different action strategies. (A) Acquisition of lever-pressing for ChR D_1_-Cre animals (n = 6) and YFP controls (n = 6). (B) Acquisition of lever-pressing for ChR D_2_-Cre animals (n = 10) and YFP controls (n = 9). (C) Difference in pressing from the first to the last day of training for ChR and YFP D_1_-Cre, for active and inactive levers. (D) Difference in pressing from the first to the last day of training for ChR and YFP D_2_-Cre, for active and inactive levers. (E,F) Probability of transition from an active lever press to a subsequent active lever press (versus an inactive press) for (E) ChR D_1_-Cre and (F) ChR D_2_-Cre. (G,H) Contingency degradation and reinstatement for (G) D_1_-Cre and (H) D_2_-Cre. Mean ± s.e.m. plotted in all graphs; LD: last day of training; CD: contingency degradation day; R: reinstatement day. ^∗^ denotes p < 0.05.

D1-Cre animals acquired lever-pressing rapidly, and pressed the active significantly more than the inactive lever (Figure 1A, F3,20 = 21.21, P < 0.0001; Figure 1C, first versus last day of active lever with ChR: P < 0.0001). On the other hand, D2-Cre animals expressing ChR were slower in acquisition, and showed a significant increase in lever-pressing for both levers (Figure 1B, F3,34 = 3.111, P = 0.0390; Figure 1D, first versus last day for both active and inactive lever with ChR: P < 0.05). This difference does not stem from different numbers of pairings between action and reinforcer in D1- and D2-cre animals, because the same result was observed when matching the number of reinforcers between groups (Supplemental Figure S1D,E).

These data suggest stimulation of both dMSNs and iMSNs in DLS is reinforcing and not aversive, but leads to the development of different action strategies. To better characterize this dichotomy, we calculated the probability of pressing the active versus the inactive lever. D1-Cre animals expressing ChR showed a steady increase in the probability of pressing the active lever (F1,10 = 688.3, P < 0.0001, Supplemental Figure S1F), while D2-Cre animals converged to a similar probability of pressing either lever with training. To further investigate if this pressing pattern resulted from action generalization, or from avoidance of the active lever by shifting to the inactive after an active press, we calculated the probability of an active-to-active transition (or conversely, an active-to-inactive transition, Figure 1E,F). D1-Cre animals reached a high probability of making an active press following an active one (F1,10 = 310.9, P < 0.0001, Figure 1E). D2-Cre animals presented a slight but significantly higher probability of pressing the active lever after an active press throughout training (F1,18 = 13.38, P = 0.0018, although close to chance, Figure 1F), indicating that D2-Cre mice were not just shifting to the inactive lever after an active lever press.

These data suggest that iMSN self-stimulation leads to more generalization between levers, which is consistent with the role of DLS in generalization and habit learning [5]. To evaluate if the actions of both groups were equally sensitive to action-stimulation contingency, we performed a contingency degradation (CD) session, where light stimulation was non-contingent upon lever-pressing. D1-Cre animals decreased the number of presses during CD (Figure 1G, Last day versus CD for ChR-A animals: P < 0.001), and resumed their lever-pressing behavior during contingency reinstatement (CD versus reinstatement for ChR-A animals: P < 0.01). D2-Cre animals, on the other hand, presented no changes in pressing during CD (Figure 1H), suggesting that pressing in these animals is less sensitive to action-stimulation contingency.

Here we show that self-stimulation of both striatonigral and striatopallidal DLS neurons is sufficient to positively reinforce actions, but that stimulation of each pathway supports the learning of different action strategies. While dMSN stimulation resulted in rapid task acquisition, selective pressing of the active lever, and sensitivity to changes in contingency, iMSN self-stimulation resulted in slower lever-press acquisition, generalized pressing between active and inactive levers, and insensitivity to changes in contingency. Since activity in both pathways precedes lever-pressing [6], plasticity associated with instrumental learning could be occurring at recently active corticostriatal synapses (and be different for dMSN and iMSN synapses). Alternatively, stimulation of MSNs could specifically select inputs onto cortical neurons that were previously active through the cortico-basal ganglia-thalamocortical loop.

These results suggest that pairing activation of DLS dMSNs with an action supports goal-directed learning, while pairing activation of DLS iMSNs with an action supports the formation of stimulus-response habits [5]. These conclusions are consistent with the role of long-lasting plasticity of glutamatergic inputs into DLS striatopallidal neurons in habit formation and skill consolidation 4, 8. They also raise the possibility that DLS might not be homogenously involved in habit formation; direct and the indirect pathways in DLS could support different action strategies and compete for action control.

These results are also consistent with involvement of both striatal projection pathways in action selection, dMSNs supporting the execution of the desired actions, and iMSNs inhibiting the execution of competing actions 6, 9. These roles may be different in DMS, where striatonigral and striatopallidal neurons seem to have opposite roles in reinforcement [2]. But it could be that other factors, such as stimulation protocol (Supplemental Figure S2A,B), or the actions/task used, explain the differences. Still, it is clear from these results that self-stimulation of iMSNs in DLS is not aversive. In this context, it is interesting to note that optogenetic stimulation of iMSNs leads to the activation of a subset of cortical M1 neurons [10], and that inactivation of iMSNs does not necessarily increase basal ganglia output activity [7], underscoring that the functional organization of basal ganglia is more complex than classically proposed.

## Supplemental Information

Document S1. Two figures, supplemental statistics for Figure 1, and experimental procedures
