# Supplementary material for: Direct and indirect dorsolateral striatum pathways reinforce different action strategies
Source: Curr Biol. 2016 Apr 4;26(7):R267–9. doi: 10.1016/j.cub.2016.02.036 (PMC4826435; doi:10.1016/j.cub.2016.02.036)
Supplement: Document S1. Two figures, supplemental statistics for Figure 1, and experimental procedures [file mmc1.pdf]

Supplemental Information

Direct and indirect dorsolateral striatum pathways reinforce different action strategies

Ana M. Vicente*, Pedro Galvão-Ferreira*, Fatuel Tecuapetla, and Rui M. Costa

Supplemental statistics for Figure 1:

Figure 1A - Main effect of D_1 training $F_{14,280}$=5.143, P<0.0001; lever and ChR effect $F_{3,20}$=21.21, P<0.0001; Interaction $F_{42,280}$=4.760, P<0.0001.

Figure 1B - Main effect of D_2 training $F_{31,1054}$=1.516, P=0.0355; lever and ChR effect $F_{3,34}$=3.111, P=0.0390; Interaction $F_{93,1054}$=1.093, P=0.2643.

Figure 1C - Main effect of D_1 training $F_{1,20}$=53.18, P<0.0001; lever and ChR effect $F_{3,20}$=45.38, P<0.0001; Interaction $F_{3,20}$=50.14, P<0.0001. Post hoc ChR active first day versus ChR active last day: P<0.0001.

Figure 1D - Main effect of D_2 training $F_{1,34}$=8.282, P=0.0069; lever and ChR effect $F_{3,34}$=3.858, P=0.0177; Interaction $F_{3,34}$=3.442, P=0.0274. Post hoc ChR active first day versus ChR active last day: P<0.05; ChR inactive first day versus ChR inactive last day: P<0.05.

Figure 1E - Main effect of D_1 training $F_{14,140}$=1.752x10^{-14}, P>0.9999; lever effect $F_{1,10}$=310.9, P<0.0001; Interaction $F_{14,140}$=7.485, P<0.0001. Post hoc p(active after active) versus p(inactive after active): P<0.0001 sessions 3-15.

Figure 1F - Main effect of D_2 training $F_{31,558}$=5.696x10^{-15}, P>0.9999; lever effect $F_{1,18}$=13.38, P=0.0018; Interaction $F_{31,558}$=1.176, P=0.2362. Post hoc p(active) versus p(inactive): P<0.01 for session 13; P<0.05 for session 32.

Figure 1G - Main effect of D_1 contingency degradation $F_{2,20}$=6.410, P=0.0071; lever effect $F_{1,10}$=45.68, P=0<0001; Interaction $F_{2,20}$=5.687, P=0.0111. Post hoc ChR active Last day versus ChR active CD: P<0.001; ChR active CD versus ChR active R2: P<0.01.

Figure 1H - Main effect of D_2 contingency degradation $F_{2,36}$=0.09552, P=0.9091; lever effect $F_{1,18}$=3.295, P=0.0862; Interaction $F_{2,36}$=1.331, P=0.2769.

Supplemental experimental procedures:

Animals. All procedures were reviewed and performed in accordance with the Champalimaud Centre for the Unknown Ethics Committee guidelines, and approved by the Portuguese Veterinary General Board (Direcção Geral de Veterinária, approval 0421/000/000/2014). Male mice between 2 and 5 months of age, resulting from the backcrossing of BAC transgenic mice into Black C57BL for at least 8 generations (which express the Cre recombinase under the control of the dopamine D1a (EY217 line) or D2 (ER43 line) receptor promoters) [S1] were used in this study. These lines were chosen because their expressions are more restricted to striatum, to avoid possible contaminations from potential cortical stimulation. After surgery mice were housed individually under a 12 hours light/dark cycle. Experiments were performed on the light cycle.

Surgery and Histology. Surgeries were performed under anesthesia using a mix of oxygen (1 – 1.5 l/min) and isoflurane (1 – 3 %). Each animal was bilaterally injected with 1.5 µl of viral solution in dorsolateral striatum (DLS – anterior-posterior: 0.38 mm from bregma, mediolateral: 2.5 mm from bregma; dorsoventral: 2.2 mm from the brain surface) [S2],using a glass pipette, by pressure (nanojet II from Drummond Scientific, with 4.6 nl pulses at a rate of 0.4 Hz). The viruses injected were AAV2/1.EF1a.DIO.hChR.eYFP (University of North Carolina, titer 5.58×10^{12}) for ChR animals, and AAV2/1.EF1a.DIO.eYFP (University of North Carolina, titer 1.85×10^{12}) for control animals. For optical stimuli delivery, fiber optics (200 µl diameter, NA=0.22) [S3] were implanted at the site of injection, 2.0 mm from the brain surface. Animals were sacrificed after completion of the behavior. Following anesthesia, both control and ChR groups were perfused with saline and paraformaldehyde (4%). Their brains were removed for histological analysis and sectioned in 50 µm coronal slices (Leica vibratome). Both placement of fibers and spread of injection were investigated using a Zeiss AxioImager.M2 widefield fluorescence scanning microscope.

Behavioral procedures. 2 weeks after surgery, the behavior of the animals was tested in an instrumental task. Training took place in behavioral chambers (MED-PC, dimensions 23 cm x 20 cm x 19.5 cm – W x D x H) placed in sound attenuating boxes. Each chamber was equipped with a food magazine, a house light place on the wall on the left of the magazine and two retractable levers, one on each side of the magazine. MED-PC IV software was used to control the equipment and record lever presses, head entries to the magazine and laser on-set. Master8 software was used to drive the laser pulses, and Labview was used to

record the behavior of the animals in video. Optical stimulation was delivered to both ChR and control animals with implantables fibers [S3] connected to a rotatory joint (Doric Lenses) coupled to a 200 mW and 473 nm laser (Shanghai Dream Lasers Technology Co., Ltd). Each stimulation consisted of 10 ms pulses delivered at 5 Hz [S4] during 2 seconds, driven by an acousto-optic modulator (AA Opto Electronic) receiving TTL pulses from a Master8 stimulator (A.M.P.I.). The power of the laser was adjusted ex-vivo to be 5-10 mW per hemisphere at the tip of a reference fiber. During training, a session started with the illumination of the house light and extension of both levers. One lever was the active lever (AL) and one was the inactive lever (IL). The levers used as AL and IL were counterbalanced within groups. Optical stimuli to the dorsolateral striatum (DLS) were delivered contingently upon pressing the AL. Animals were trained one session a day, during 30 minutes each and no limit on the number of stimuli, on a continuous reinforcement (CRF) schedule, where each press led to one stimulus. For D_2 animals, animals were trained on CRF for at least 32 days. D_1 animals were trained for at least 15 days. After acquisition mice received contingency degradation training. In each CD session (30 minutes long) laser onset happened at a random time schedule and non-contingent upon lever press, i.e. independent of the animals' behavior. The number of laser stimulations was yoked independently for each animal, based on their average presses on the last 3 days of training (thus, with the same probability of reinforcement per unit of time as during training). The D_1 group had 1 session of CD, while the D_2 group had 2 session of CD (to guarantee that indeed they were not sensitive to CD). As previously shown [S5], insensitivity to contingency degradation in iMSN-stimulated animals is unlikely to be due to a floor effect. Following CD animals received a session of reinstatement, equal to the CRF training sessions before CD.

In Vivo Recordings. In vivo recordings were performed with movable bundle of 16 platinum-coated tungsten microwires with coupled guide cannulas to introduce a fibre optic 200–300 µm away from the tip of the electrodes (Innovative-Neurophysiology). Neural activity and light stimulation timestamps were recorded with a Cerebrus recording system (Blackrock Microsystems). After recording units were offline sorted (Offline Sorter, Plexon Inc.) and time stamps and waveforms were exported to MATLAB for further analyses.

Statistical analysis. Statistical analyses were performed using GraphPad Prism 6 (GraphPad Software Inc., CA, USA). Repeated measures ANOVA were used to evaluate acquisition of lever presses and contingency degradation, followed by post hoc analyses using the

Dunnet's test and the Sidák correction when appropriate. Planned paired t-tests were used for the comparisons between active and inactive levers when matching for reinforcers. Statistical significance was set at alpha=0.05. Results were presented as mean ± SEM.

Supplemental references for methods:

S1 Gong, S., Doughty, M., Harbaugh, C.R., Cummins, A., Hatten, M.E., Heintz, N., and Gerfen, C.R. (2007). Targeting Cre recombinase to specific neuron populations with bacterial artificial chromosome constructs. J Neurosci *27*, 9817–9823.

S2 Paxinos, G., and Franklin, K.B.J. (2001). Mouse Brain in Stereotaxic Coordinates, Second Edition (San Diego: Academic Press).

S3 Sparta, D.R., Stamatakis, A.M., Phillips, J.L., Hovelsø, N., van Zessen, R., and Stuber, G.D. (2012). Construction of implantable optical fibers for long-term optogenetic manipulation of neural circuits. Nat Protoc *7*, 12–23.

S4 Tecuapetla, F., Matias, S., Dugue, G.P., Mainen, Z.F., and Costa, R.M. (2014). Balanced activity in basal ganglia projection pathways is critical for contraversive movements. Nat Commun *5*, 1-10.

S5 Hilário, M.R.F., Clouse, E., Yin, H.H., and Costa, R.M. (2007). Endocannabinoid signaling is critical for habit formation. Front Integr Neurosci *1*, 1–12.

Supplemental figures:

Figure S1.

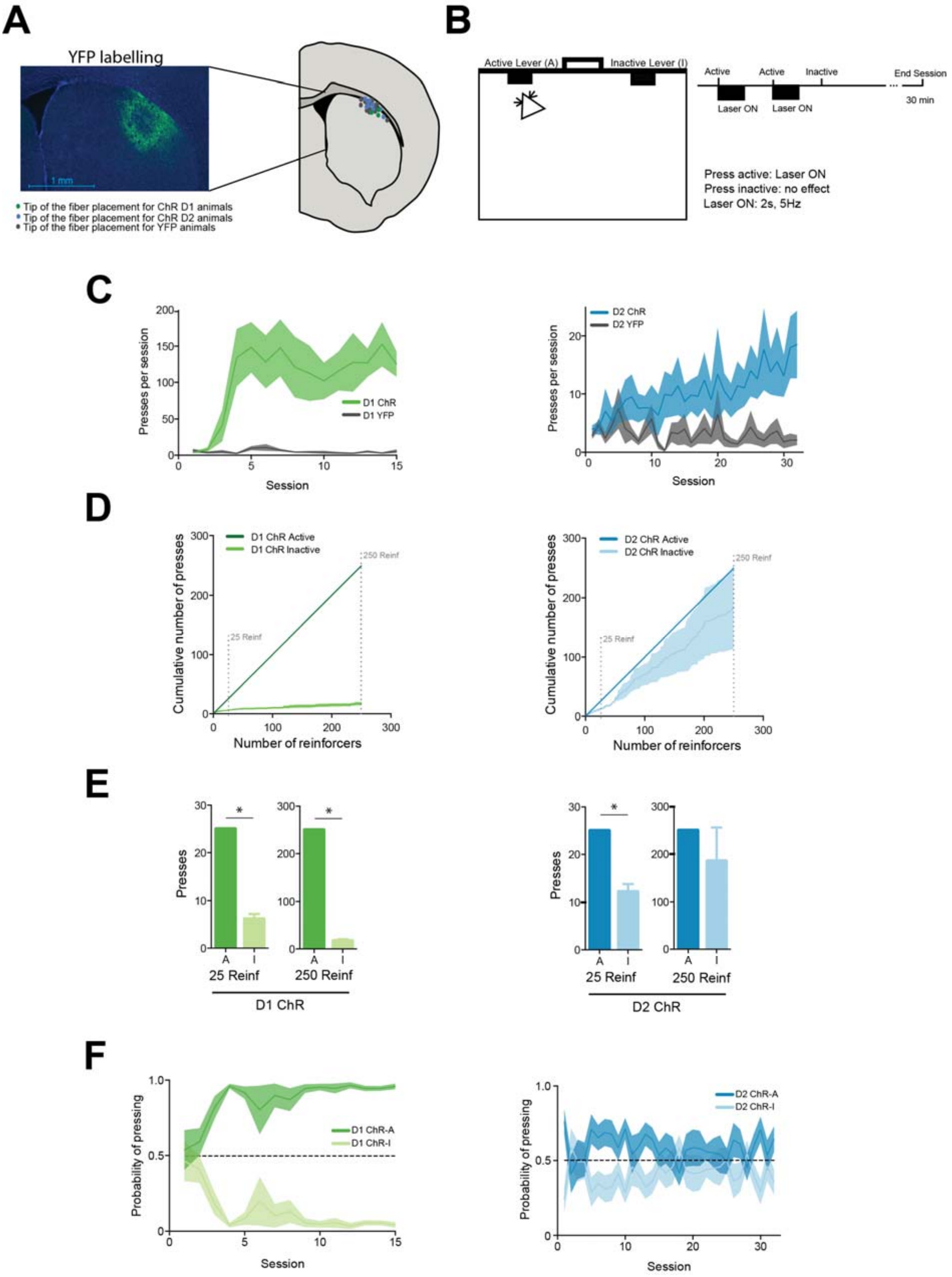

Figure S1. (A) Schematics and representative histology slice of injection and fiber placement sites in DLS. **(B)** Schematics of the operant box and the behavioral paradigm. **(C)** Acquisition of lever-pressing for ChR D_1-Cre animals (n=6) and YFP controls (n=6) (left panel, main effect of D_1 training $F_{14,140}=4.987$, $P<0.0001$; ChR effect $F_{1,10}=20.67$, $P=0.0011$; Interaction $F_{14,140}=4.883$, $P<0.0001$) and acquisition of lever-pressing for ChR D_2-Cre animals (n=10) and YFP controls (n=9) (right panel, main effect of D_2 training $F_{31,527}=1.120$, $P=0.3026$; ChR effect $F_{1,17}=5.845$, $P=0.0271$; Interaction $F_{31,527}=1.505$, $P=0.0411$). **(D)** Cumulative active and inactive presses for D_1-cre and D_2-cre for the first 250 reinforcers earned. **(E)** Cumulative number of presses for 25 and 250 reinforcers for D1-cre and D2-cre (Paired, two-tailed t-tests: *<0.005). **(F)** Probability of pressing the active versus the inactive lever for D_1-Cre animals (Main effect of D_1 training $F_{14,140}=3.447 \times 10^{-14}$, $P>0.9999$; lever effect $F_{1,10}=688.3$, $P<0.0001$; Interaction $F_{14,140}=7.367$, $P<0.0001$. Post hoc p(active) versus p(inactive): $P<0.0001$ sessions 3-15) and D_2-Cre animals (Main effect of D_2 training $F_{31,558}=5.904 \times 10^{-15}$, $P>0.9999$; lever effect $F_{1,18}=6.961$, $P=0.0167$; Interaction $F_{31,558}=1.903$, $P=0.0026$. Post hoc p(active) versus p(inactive): $P<0.01$ for session 1).

Figure S2

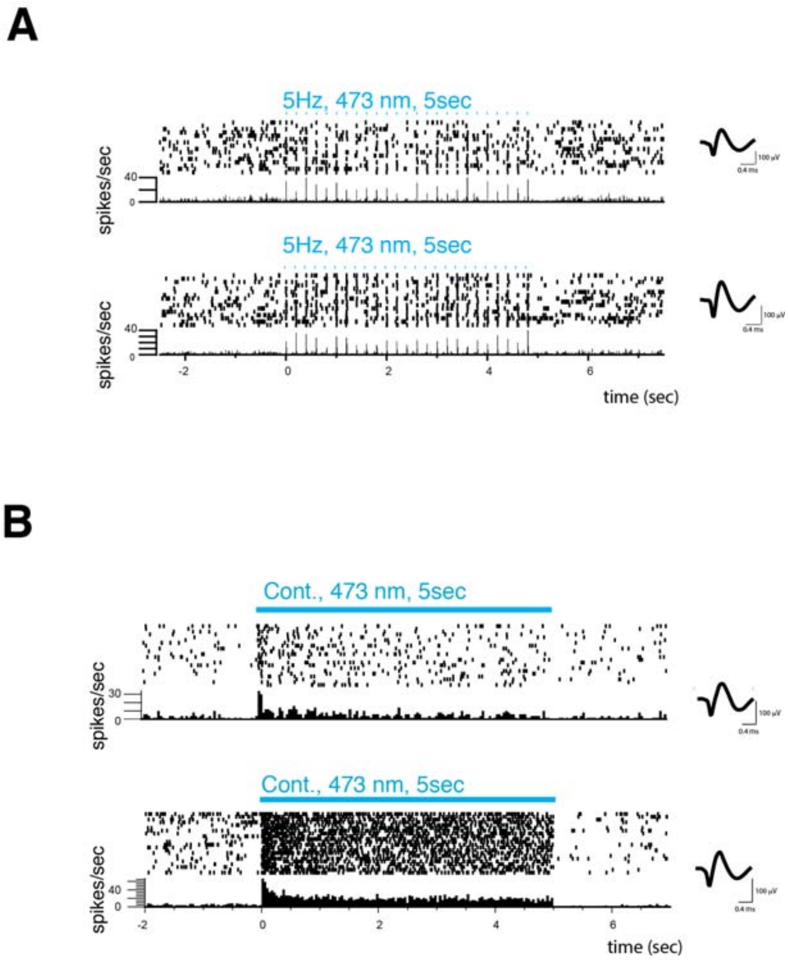

Figure S2. (A,B) Examples of peri-event histograms from individual MSN neurons expressing channelrodhopsin recorded in vivo and aligned to the on-set of a 473 nm blue laser stimulation, for D1-Cre and D2-Cre mice. **(A)** Stimulation with pulses of 5Hz, during 5 seconds, results in further biasing of ongoing activity. **(B)** Continuous light stimulation during 5 seconds leads to an initial peak of activation followed by less activation. We therefore chose to use 5Hz stimulation in this study, to avoid any nonlinear effects of continuous stimulation.

Author contributions:

Conceptualization, A.M.V., P.G.F. and R.M.C.; Methodology, A.M.V., P.G.F., F.T. and R.M.C.; Investigation, A.M.V., P.G.F., F.T. and R.M.C.; Writing –Original Draft, A.M.V., P.G.F. and R.M.C.; Writing –Review & Editing, A.M.V., P.G.F. and R.M.C.; Funding Acquisition, R.M.C.; Resources, A.M.V., P.G.F. and R.M.C.; Supervision, R.M.C.
